# Design of synthetic activators for programmable gene expression

**DOI:** 10.64898/2026.10.19.760300

**Published:** 2026-10-20

**Authors:** Wei Chen, Ruopu Jiao, Brian Coventry, Xiangli Qu, Magnus Bauer, Long Tran, Tabitha Tcheau, Kejia Wu, Zihao Song, Nicole Roullier, Xinting Li, Cameron Glasscock, Isaac B. Hilton, David Baker

## Abstract

Transcription factors control gene expression by recruiting transcriptional coactivators through intrinsically disordered transcriptional activation domains (TADs), whose weak and promiscuous interactions make it difficult to establish quantitative relationships between coactivator recruitment and transcriptional output, and to precisely modulate gene expression. To overcome this limitation, we set out to design coactivator-recruiting proteins using AlphaFold3-predicted TAD-coactivator interactions as a guide. We developed a high-throughput functional screening platform in mammalian cells and identified over 300 well folded nanomolar affinity binders to the transcription coactivators Mediator (multiple subunits), BRD4, NCOA, and p300; targeted interface mutations enabled systematic tuning of their binding affinities. Using a panel of p300 binders spanning nanomolar to micromolar affinities, we found an ultrasensitive relationship between coactivator-binding affinity and transcriptional output, with a sharp transcriptional activation threshold at ∼500 nM. Incorporating p300 binder designs into CRISPRa yielded compact synthetic effectors that drove stronger activation than VPR across multiple genomic loci with minimal detectable cytotoxicity. Combining p300- and Mediator-specific binders further enhanced transcription, revealing functional synergy between these coactivators. Together, our work establishes a quantitative framework linking coactivator-binding affinity to transcriptional output and provides compact synthetic effectors for programmable gene activation.

